# Deciphering the Infectious Process of *Colletotrichum lupini* in Lupin through Transcriptomic and Proteomic Analysis

**DOI:** 10.3390/microorganisms8101621

**Published:** 2020-10-21

**Authors:** Guillaume Dubrulle, Adeline Picot, Stéphanie Madec, Erwan Corre, Audrey Pawtowski, Riccardo Baroncelli, Michel Zivy, Thierry Balliau, Gaétan Le Floch, Flora Pensec

**Affiliations:** 1 Laboratoire Universitaire de Biodiversité et Écologie Microbienne, ESIAB, Université de Brest, F-29280 Plouzané, France; guillaume.dubrulle@outlook.com (G.D.); adeline.picot@univ-brest.fr (A.P.); audrey.pawtowski@univ-brest.fr (A.P.); gaetan.lefloch@univ-brest.fr (G.L.F.); 2 CNRS, IRD, Ifremer, LEMAR, Université de Brest, F-29280 Plouzané, France; stephanie.madec@univ-brest.fr; 3 Station Biologique de Roscoff, FR2424 CNRS Sorbonne Université, Place Georges Teissier, 29680 Roscoff, France; corre@sb-roscoff.fr; 4 Instituto Hispano-Luso de Investigaciones Agrarias (CIALE), University of Salamanca, Calle del Duero 12, 37185 Villamayor (Salamanca), Spain; riccardobaroncelli@gmail.com; 5 INRAE le Moulon, Plateforme PAPPSO, ferme du Moulon, 91190 Gif-sur-Yvette, France; michel.zivy@inrae.fr (M.Z.); thierry.balliau@inrae.fr (T.B.)

**Keywords:** anthracnose disease, transcriptome, proteome, pathogenicity factors

## Abstract

The fungal phytopathogen *Colletotrichum lupini* is responsible for lupin anthracnose, resulting in significant yield losses worldwide. The molecular mechanisms underlying this infectious process are yet to be elucidated. This study proposes to evaluate *C. lupini* gene expression and protein synthesis during lupin infection, using, respectively, an RNAseq-based transcriptomic approach and a mass spectrometry-based proteomic approach. Patterns of differentially-expressed genes in planta were evaluated from 24 to 84 hours post-inoculation, and compared to in vitro cultures. A total of 897 differentially-expressed genes were identified from *C. lupini* during interaction with white lupin, of which 520 genes were predicted to have a putative function, including carbohydrate active enzyme, effector, protease or transporter-encoding genes, commonly described as pathogenicity factors for other *Colletotrichum* species during plant infection, and 377 hypothetical proteins. Simultaneously, a total of 304 proteins produced during the interaction were identified and quantified by mass spectrometry. Taken together, the results highlight that the dynamics of symptoms, gene expression and protein synthesis shared similarities to those of hemibiotrophic pathogens. In addition, a few genes with unknown or poorly-described functions were found to be specifically associated with the early or late stages of infection, suggesting that they may be of importance for pathogenicity. This study, conducted for the first time on a species belonging to the *Colletotrichum acutatum* species complex, presents an opportunity to deepen functional analyses of the genes involved in the pathogenicity of *Colletotrichum* spp. during the onset of plant infection.

## 1. Introduction

The distribution of *Colletotrichum* spp. is global. This anthracnose agent has been classified in the top 10 fungal pathogens of scientific and economic importance [1]. Within this genus, *Colletotrichum lupini,* responsible for lupin anthracnose, belongs to the *Colletotrichum acutatum* species complex which includes, so far, 34 species [2,3]. Infected lupin fields with *C. lupini* may result in significant yield loss. Gondran et al. (1997) [4] demonstrated that 0.1% of infected seeds could provoke more than 50% yield loss. The history, importance and epidemiology of this little-known pathogen, which has caused severe disease outbreaks over recent decades in Europe and Australia, were reviewed in 2016 by Talhinhas et al. [5]. Infected seeds are the primary inoculum source. After germination, lesions appear on plants, and conidia are produced and spread to neighboring seedlings. Secondary inoculations can occur during the plantlet and flowering stages, generating infections of pods and seeds [6,7]. The lack of resistance of lupin, mainly to *C. lupini,* limits the use of this leguminous crop in rotation systems [5]. The development of successful solutions for crop protection requires new insights into the pathogenicity mechanisms of this fungus.

*Colletotrichum* spp. is characterized by a wide diversity of important traits such as host range, trophic mode and reproductive strategy [1,3,8]. Among these phytopathogenic fungi, several lifestyles have been identified, ranging from necrotrophy to hemibiotrophy and endophytism. Some were identified as necrotrophic pathogens with a quiescent phase, such as *C. gloeosporioides* on tomato plants [9], but most species are hemibiotrophic [1], and typically use a melanized appressorium to penetrate host tissues [10], and start a biotrophic phase once inside the plant cells, before switching to a necrotrophic phase [8]. The molecular mechanisms underlying these diverse infectious processes have been studied for several *Colletotrichum* species using transcriptomic analysis such as *C. higginsianum* on *Arabidopsis thaliana* and *C. graminicola* on maize leaves [11], *C. fructicola* on strawberry plants [12] or *C. falcatum* on sugarcane [13]. 

During the first phase of infection, i.e., from penetration to the biotrophic phase, which generally occurs in the first two days postinfection, gene encoding effectors are particularly expressed in several *Colletotrichum* species such as *C. gloeosporioides*, *C. lentis*, *C. fructicola*, *C. higginsianum* and *C. graminicola* [11,12,14,15]. These small cysteine-rich secreted proteins are characterized by the absence of transmembrane helices (TMH) and glycosylphosphatidylinisotol (GPI) anchor addition sites, and show no homology to proteins outside the genus [11]. Effectors such as CgDN3 of *C. gloeosporioides* [16], ChEC36 and ChEC6 of *C. higginsianum* [14] were found to be expressed in the biotrophic stage, including in unpenetrated appressoria in planta, notably for the two latter [14,17]. Among the *Colletotrichum* studied so far, the biotrophic phase is also characterized by a higher expression of genes associated with secondary metabolism and genes encoding carbohydrate active enzymes (CAZymes) [11,12,18,19]. 

The switch to necrotrophy generally leads to a completely different expression pattern, in particular, a higher expression of genes encoding hydrolases, proteases and transporters [9,11,12,18]. A great diversity of CAZymes and cellulases are also known to be secreted and enable pathogens to degrade plant tissues and absorb intracellular nutrients, ultimately leading to necrotic lesions in the necrotrophic phase [20]. Nutrients released by the infected tissue are taken up by transmembrane transporters such as oligopeptide transporters, ABC transporters and those belonging to the facilitator superfamily, some of which are known to be involved in the pathogenicity of *Colletotrichum* spp. [21,22,23]. Necrosis- and ethylene-inducing proteins (NEPs) which act as elicitors of the hypersensitive response in plants also belong to pathogenicity factors produced during the necrotrophic phase, and are widely distributed across diverse groups of phytopathogens [24].

Interestingly, although typical pathogenicity traits were found to be very similar among *Colletotrichum* species, diversity in molecular expression patterns was observed during infection. A comparative study by O’Connell et al. (2012) [11] showed that secondary metabolism gene clusters, present in both *C. higginsianum* and *C. graminicola*, were remarkably activated in the former while remaining nonexpressed in the latter at all stages of infection in maize leaves. These authors also found that the number of putative effectors in the transcriptome varied, ranging from 275 in *C. higginsianum* to 177 in *C. graminicola*. This diversity in the molecular pattern of infection is probably linked to the ability of some species to infect a broad range of hosts compared to others, limited to few or specific to one host [11,18].

To date, the proteome or secretome of *Colletotrichum* spp. during interaction with their host has been poorly studied. Among the studies carried out, a quantitative analysis of proteins synthesized during appressorium formation of *C. acutatum* infecting strawberries was performed by two-dimensional gel electrophoresis and mass spectrometry (LC-MS/MS). The authors demonstrated that nitrogen-limiting conditions induced changes in protein abundance during appressorium formation, such as activation of the glyoxylate bypass, and that the generation and elimination of reactive oxygen species (ROS) were key factors for germinating conidia under nutritional deprivation [25]. The proteome of the same species was also described during conidial germination using two-dimensional electrophoresis combined with MALDI-TOF/TOF mass spectrometry. The authors identified 245 unique proteins associated with germination, in comparison to ungerminated conidia, some of which were associated with pathogenesis [26]. More recently, the proteome of *C. lini*, an industrial strain involved in the synthesis of an oral contraceptive, was studied via an iTRAQ-based quantitative proteomic analysis in response to ethanol induction. This method showed that different proteins were differentially regulated under ethanol induction, and allowed the authors to explore the molecular mechanisms involved [27]. Numerous studies have also identified some proteins associated with the pathogenicity of *Colletotrichum* spp., in particular, the scytalone dehydratase associated with the melanization and/or the kelch repeat proteins and the Zn(2)-Cys(6) transcription factor associated with the synthesis of appressoria via mutant generation [28,29,30,31]. Other approaches using random insertional mutagenesis evidenced a large number of proteins involved in the pathogenicity process of *C. higginsianum* [32,33], or *C. gloeosporioides* [34], but not in interaction with plants.

Here, we report the transcriptome and proteome analysis of *C. lupini* infecting lupin using RNAseq and a nLC Q-exactive Orbitrap mass spectrometry. Lupin-infected samples for gene expression were collected at five time-points from 24 h post-inoculation (hpi) to 84 hpi. Sampling times for proteomic analysis were delayed by 12 h in order to take into account the duration of the protein translation steps. This work enabled us to show the specific kinetics of expression and synthesis of genes and proteins associated with the pathogenicity of *C. lupini* infecting white lupin (*Lupinus albus)*. Virulence markers characteristic of hemibiotrophic agents were encountered during infection, but some poorly described or unknown genes were also specifically associated with early or late stages of infection, suggesting they could be interesting candidates to investigate.

## 2. Materials and Methods

### 2.1. Fungal Isolate and Plant Material

#### 2.1.1. Fungal Culture Condition and Inoculum Preparation

The RB221 strain of *C. lupini* (IMI 504893; UBOCC-A-117274) was isolated from a lupin crop in Brittany in 2014. The genome sequence of this strain was obtained using Illumina GAII and HiSeq 4000 instruments, and also using Pacific Biosciences technology (PacBio, Menlo Park, CA, USA). The inoculum of the strain RB221 was prepared from a two week-old PDA culture at 25 °C, by adding 1.5 mL of distilled sterile water to the culture. Spore concentration was adjusted to 1 × 10^4^ spores·mL^−1^. Five microliters of the spore suspension was used to inoculate sterilized lupin boiled seeds (adapted from Saubeau et al. (2014) [35]) placed on 1.3% water-agar Petri plates. After one-week of incubation at 25 °C, spores were harvested by shaking the infected lupin seeds into 3–4 mL of distilled water, and the spore inoculum was adjusted to 1 × 10^7^ spores·mL^−1^. For each biological repetition, a new fungal culture and inoculum was prepared. 

#### 2.1.2. Plant Growth

The lupin variety used for inoculation was a spring variety, named Feodora (Jouffray-Drillaud, Cissé, France). Seeds were disinfected for one minute in 70% ethanol, then rinsed three times with sterile distilled water before being placed on water-agar media and incubated at 25 °C in darkness for three days until germination. Inoculated plants were grown at 25 °C day and night, with 16 h of light and 8 h of darkness, and 60% humidity. Plants were watered every two days throughout the experiment. Symptoms were evaluated on all inoculated plants by measurement of the lesion length whenever visible. An ANOVA was performed to study the influence of biological repetition on necrosis length, using the RStudio software (version 3.2.5). Differences between variables were determined with multiple comparisons tests with the post hoc Tukey HSD method. The level of significance was set at α = 0.05.

#### 2.1.3. Inoculation and Incubation Conditions

Twenty microliters of the inoculum adjusted at 1 × 10^7^ spores·mL^−1^ were dropped at the base of the radicle emerging from the seed. Inoculated seedlings were put in Jiffy^®^ pots (peat pellets, 41 mm diameter) (Jiffy Products International BV, Zwijndrecht, Netherlands) for 24, 36, 48, 60, 72, 84 and 96 hpi. Sampling times were selected to target the first stages of the infection, the putative switch between biotrophic and necrotrophic phases and the necrotrophic phase. At 24, 48, 60, 72 and 84 hpi, 10 inoculated-seedlings were used for RNAseq analysis, while at 36, 60, 72, 84 and 96 hpi, 10 additional ones were used for proteomic analysis. Four biological repetitions were performed (except at 96 hpi, when three biological repetitions were done). Seedlings inoculated with water were used as negative controls. 

Control conditions for proteomic and transcriptomic analyses were performed in vitro in a Czapek Dox liquid medium with 0.5 g·L^−1^ of sucrose instead of 30 g·L^−1^ (adapted from BD Difco™ CzaPEK-Dox Broth composition, BD, Franklin Lakes, NJ, USA) to simulate the low availability of carbohydrates on plant surface during fungal infection and germination. For each of the four repetitions, five Erlenmeyer flasks containing 100 mL of liquid medium were inoculated with 2 × 10^6^ spores of *C. lupini*, as described previously. Flasks were then incubated for 24 h under agitation at 120 rpm at 25 °C before filtration for RNA extraction.

Microscope observations of morphological structures of *C. lupini* infecting plants were obtained from stem sections of two week-old lupin plants grown in vitro on Farheus medium under the same photoperiod as described previously. Under sterile conditions, cut ends were sealed with molten paraffin wax and droplets of spore suspensions at 10^5^ spores·mL^−1^ were placed at intervals along the hypocotyl. After 12, 24, 48 and 72 hpi, epidermal strips were carefully removed, colored by lactophenol cotton blue and then placed in a drop of water for microscope observation.

### 2.2. Preparation of RNA Samples for RNA Sequencing Analysis

#### 2.2.1. Extraction of *C. lupini* RNA from Pure Culture

The extraction of RNA from pure cultures of *C. lupini* was performed from mycelium grown in Czapek Dox media with 0.5 g·L^−1^ of sucrose as described above. Mycelium was filtered through 0.45 µm cellulose acetate filter by Büchner filtration method and frozen in liquid nitrogen. One hundred milligrams of frozen mycelium was ground in a Mixer Mill (Retsch, MM400, Éragny, France) four times at a frequency of 30 Hz for one minute in lysing matrix A tube (MP biomedicals, Santa Ana, CA, USA). RNA extraction was performed with the RNeasy Plant Mini Kit (Qiagen, Hilden, Germany) following the manufacturer’s instructions. Samples were shaken using Mixer Mill (Retsch, MM400, Éragny, France) three times at a frequency of 30 Hz for one minute, after adding lysing buffer RLT. A DNase digestion step with RNase-Free DNase Set (Qiagen, Hilden, Germany) was added after the nucleic acid precipitation step and following the manufacturer’s instructions. 

#### 2.2.2. Extraction of Total RNA from Inoculated Plants

At each time-point, infected plants were removed from Jiffy^®^ pots. Approximately 100 mg of the radicle of ten plants inoculated in the same conditions were cut with a scalpel around the inoculation or necrosis area. The ten pieces of plants were pooled together and frozen in liquid nitrogen. Samples were first ground using mortar and pestle, then using gentleMACS™ Dissociator (Miltenyi Biotec GmbH, Bergisch Gladbach, Germany) with gentleMACS™ C tubes three times at 4000 rpm for 20 s. The RNA extraction was performed with the RNeasy Plant Mini Kit (Qiagen, Hilden, Germany) following the manufacturer’s instructions. The total volume of lysing buffer RLT was adjusted to 1 g of ground plant corresponding to the pool of ten plants. A DNase digestion step with RNase-Free DNase Set (Qiagen, Hilden, Germany) was added after nucleic acid precipitation. Total RNA quality and quantity control was validated using Nanodrop1000 (Thermo Fischer Scientific, Waltham, MA, USA) and Agilent 2100 Bioanalyzer system analysis (Agilent technologies, Santa Clara, CA, USA) with RNA 6000 Nano LabChip^®^ Kit according to the manufacturer’s instructions. The samples that were examined were expected to have a RIN (RNA Integrate Number) above eight, a total area under 18S and 25S curve above 50% and an rRNA ratio (25S/18S) above 1.8.

### 2.3. Preparation of Libraries, RNA Sequencing and Analysis

Total RNA extracted from liquid culture and inoculated plants were sent to Genome Quebec Innovation Centre (McGill University, Montreal, Canada) for sequencing. Libraries were generated from 250 ng of total RNA as follows: mRNA enrichment was performed using the NEBNext Poly(A) Magnetic Isolation Module (New England BioLabs, Ipswich, MA, USA). cDNA synthesis was achieved with the NEBNext RNA First Strand Synthesis and NEBNext Ultra Directional RNA Second Strand Synthesis Modules (New England BioLabs, Ipswich, MA, USA). The remaining steps of library preparation were done using the NEBNext Ultra II DNA Library Prep Kit for Illumina (New England BioLabs, Ipswich, MA, USA). Adapters and PCR primers were purchased from New England BioLabs. Libraries were quantified using the Quant-iT™ PicoGreen^®^ dsDNA Assay Kit (Life Technologies, Carlsbad, CA, USA) and the Kapa Illumina GA with Revised Primers-SYBR Fast Universal kit (Kapa Biosystems, Wilmington, MA, USA). Average fragment size was determined using a LabChip GX (PerkinElmer, Waltham, MA, USA) instrument.

The libraries were normalized and pooled before being denatured in 0.05N NaOH and neutralized using HT1 buffer. ExAMP was added to the mix following the manufacturer’s instructions. The pool was loaded at 200 pM on an Illumina cBot, and the flowcell was run on a HiSeq 4000 for 2 × 100 cycles (paired-end mode). A phiX library was used as a control and mixed with libraries at 1% level. The Illumina control software was HCS HD 3.4.0.38, the real-time analysis program was RTA v. 2.7.7. The bcl2fastq2 v2.20 program was then used to demultiplex samples and generate fastq reads. These data have been submitted to the ENA (European Nucleotide Archive) with the bioproject accession number PRJEB40331.

Read quality check and cleaning were performed on ABiMS Galaxy platform (galaxy.sb-roscoff.fr). Low quality reads were cleaned using Trimmomatic with the default parameters [36]. Paired reads were pseudo-aligned and quantified onto the sequenced and annotated *C. lupini* RB221 genome using Salmon package (Version 0.8.2) of Galaxy platform [37]. Genes which had a minimum of 100 counts per million (cpm) values in at least three repetitions were kept for further analysis. 

In planta, gene expression for each time post-inoculation was first compared to the liquid culture condition to identify differentially-expressed genes (DEGs) during host interaction, using DESeq2. Then, a pairwise comparison between two close time points (i.e., 84 hpi vs. 72 hpi, 72 hpi vs. 60 hpi, 60 hpi vs. 48 hpi and 48 hpi vs. 24 hpi) was performed to determine if DEGs expression was significantly different using DESeq2. DEGs were selected with a false discovery rate (FDR) below 0.05 and a Log2-Fold-Change (LFC) higher than 1 or lower than −1. It was also verified that most genes expressed in planta were also found in vitro, ensuring that important genes were not discarded by using the liquid culture condition as a point of comparison. 

A heatmap of DEGs was generated using the heatmap.2 function for the gplots R package based on normalized reads counts transformed to Z-score. Data were scaled by row, and Pearson correlation distances between genes were used for hierarchical clustering based on the expression profiles of DEGs.

### 2.4. Extraction and Digestion of Total Proteins

A pool of ten inoculated stems was ground in liquid nitrogen using a mortar and pestle. Proteins were extracted from 200 µL of stem powder using the TCA acetone protocol described in Méchin et al. (2007) [38].

Proteins were solubilized in 30 μL per mg of extract with a buffer containing 6 M urea, 2 M thiourea, 10 mM dithiothreitol (DTT), 30 mM Tris-HCl pH 8.8, and 0.1% zwitterionic acid labile surfactant (ZALS I, Proteabio, Morgantown, WV, USA) in water. Protein powders were mixed in the buffer before vortexing the tubes for 3 min. Samples were centrifuged (14,000 g, 25 min, 25 °C) and supernatants were transferred into new tubes. Protein concentrations were estimated using the plusOne 2DQuant Kit (GE Healthcare, Little Chalfont, UK), and adjusted to 3 μg·μL^−1^ prior digestion.

Digestion was performed in 0.2 mL strip tubes from 10 μL of diluted proteins representing 30 µg of proteins. Tubes were incubated for 30 min at room temperature for protein reduction by the 10 mM DTT present in the solubilization buffer. Then, 2 μL of a solution containing 300 mM of iodoacetamide (IAA) in 50 mM ammonium bicarbonate (BICA) was added, and proteins were alkylated by 1 h incubation at room temperature in the dark. After adding 90 μL of 50 mM of BICA, proteins were digested overnight with 800 ng of trypsin (Promega V5111, Promega, Fitchburg, WI, USA) at 37 °C. Trypsin digestion was stopped by adding 5.5 μL of 18.6% trifluoroacetic acid (TFA) representing 1% of the final concentration. Samples were incubated for 1 h at room temperature to allow TFA to cleave ZALS I. Peptides were desalted using C18 solid phase extraction (SPE) cartridges (strata XL 100 μm ref 8E-S043-TGB, Phenomenex, Torrance, CA, USA) as follows. The samples were first diluted in 2% acetonitrile (ACN), and 0.6% acetic acid in water (washing buffer), to a final volume of 500 μL. Then, the cartridges were conditioned with 500 μL of ACN and rinsed three times with 500 μL of washing buffer before loading the samples. Peptides were rinsed four times with 500 μL of washing buffer, and eluted two times by adding 300 μL of 40% ACN and 0.6% acetic acid in water (final pH around 2). Finally, eluted peptides were dried in a speedvac.

### 2.5. Orbitrap Mass Spectrometry and Analysis

A total of 19 protein digests were analysed with an Eksigent nlc425 nanoHPLC (SCIEX) coupled with a Q exactive+ mass spectrometer (Thermo Fisher, Waltham, MA, USA). Peptides were solubilized in 150 µL of a loading buffer containing 2% ACN and 0.1% formic acid (FA) in water.

For each injection, 4 µL (400 ng) of solubilized peptides were loaded onto a Biosphere C18 precolumn (particle size: 5 µm, pore size: 12 nm, inner/outer diameters: 360/100 µm, length: 20 mm; NanoSeparations, Nieuwkoop, Netherlands) and desalted for 4 min with the loading buffer at 7.5 µL·min^−1^. Peptides were then separated on a Biosphere C18 column (particle size: 3 µm, pore size: 12 nm, inner/outer diameters: 360/75 µm, length: 300 mm; NanoSeparations) using buffer A (0.1% FA in water) and buffer B (0.1% FA in ACN) at 300 nL·min^−1^ as follows: (i) the column was equilibrated for 9 min with 95% of buffer A and 5% of buffer B; (ii) a linear gradient from 95% of buffer A and 5% of buffer B to 65% of buffer A and 35% of buffer B was applied for 110 min; (iii) the column was regenerated with 5% of buffer A and 95% of buffer B for 5 min. Electrospray ionization was performed at 1.8 kV with an uncoated capillary probe (noncoated capillary silica tips, 360/20-10, New Objective Inc., Woburn, MA, USA). S-lens RF level was set to 50.

Data were acquired with Xcalibur v4.0 with the following data dependent steps: (1) full MS scan: 75,000 resolution, 350–1400 *m*/*z* mass range, AGC target to 3 × 106, max injection time of 250 ms; (2) MS/MS scan: 17,500 resolution, AGC Target to 1 × 105, max injection time 120 ms, isolation window 1.5 *m*/*z*, normalized collision energy 27. Step 2 was repeated for the eight most intense ions detected in step 1 with the following criteria: intensity threshold superior as 8.3 × 103, precursor charge step of 2 and 3, dynamic exclusion of 50 s, peptide match on and exclusion of isotopes. Raw data were transformed to mzXML format using msconvert (proteowizard 3.0.7069, [39]).

Data were searched with X!Tandem (version 2015.04.01.1, [40]) against the RB221 strain *C. lupini* genome, *Lupinus angustifolius* cv. Tanjil genome (AOCW00000000.1) and a homemade database containing standard contaminants. Trypsin digestion was set in strict mode with one authorized missed cleavage. Cysteine carbamidomethylation was set as a fixed modification. Methionine oxidation, protein Nter acetylation with or without excision of methionine, Nter glutamine deamidation, Nter carbamidomethyl cysteine deamidation, and Nter glutamic acid dehydration were set as potential modifications. To identify additional peptides for proteins identified after this first pass, all samples were submitted to a second pass (refine mode of X!Tandem), in which the excision of Nter peptide signal, tryptophane oxidation, glutamine and asparagine deamidation were added as potential modifications. Additional missed cleavages were authorized at this step. Protein inference was performed by using X!TandemPipeline (cpp) v0.2.18 [41] with the following parameters: peptide e-value less than 0.01, protein e-value less than 10^−5^, two identified peptide by protein. Inference was performed using all samples together. Using a reverse version of the *Colletotrichum lupini* and *Lupinus angustifolius* database as a decoy, the FDR was estimated by X!Tandem to 0.05% and 0.01% for peptide-spectrum match and protein identification, respectively.

Peptide quantification was performed on extracted ion currents (XIC) by using Masschroq 2.2.2 [42]. Only the isotope theoretically the most intense was considered for peptide quantification. Identification and raw data are publicly available at http://moulon.inra.fr/protic. To quantify proteins, we used two complementary approaches. We used XIC to quantify proteins based on peptide intensities. Only protein-specific peptides present in at least 85% of the injections and showing a significant correlation (r > 0.5) with the other peptides of the same protein were kept for further data analysis. Normalization was performed, taking into account peptide retention time as described in Lyutvinskiy et al. (2013) [43]. Only proteins quantified with at least two peptides were considered. Protein relative abundances were computed as the sum of the normalized values of the selected peptides. One-way analyses of variance were performed on log10-transformed abundance values. The resulting *p*-values were adjusted for multiple comparisons [44]. Proteins with adjusted *p*-value < 0.01 were considered as showing significant abundance variations. All data analyses were performed using the RStudio-software (version 3.2.5).

### 2.6. Transcriptome and Proteome Prediction and Analysis Pipeline

The genome of *C. lupini* RB221 was used as a reference sequence (https://mycocosm.jgi.doe.gov/Collup1/Collup1.home.html). Predicted protein sequences of DEGs and identified proteins were used to (i) predict the function, (ii) classify them into functional classes and (iii) explore the metabolism induced during the infectious process. 

Secreted proteins were determined as proteins containing a signal peptide sequence (SignalP-5.0 Server, http://www.cbs.dtu.dk/services/SignalP/, [45]), localized in the extracellular space (WoLF PSORT, https://wolfpsort.hgc.jp/, [46]), and predicted neither a transmembrane helix (TMH) domain (TMHMM server 2.0, http://www.cbs.dtu.dk/services/TMHMM/, [47]), nor a Glycosylphosphatidyl-inositol anchor (GPI anchor) domain (PredGPI, http://gpcr2.biocomp.unibo.it/gpipe/pred.htm, [48] and GPIsom, http://gpi.unibe.ch/, [49]). Candidate effectors were predicted from the set of secreted peptides showing less than 300 amino acid residues, using EffectorP 1.0 and 2.0 (http://effectorp.csiro.au/, [49]). The CAZymes were predicted using dbCAN meta server [50] with HMMER program to annotate CAZymes domain boundaries using dbCAN HMM (Hidden Markov Model) database with a cut off e-value of 1.0 × 10^−15^ and a coverage upper than 0.35. Predicted proteases were identified by the blast of protein sequences in PHMMER and MEROPS databases using a cut-off e-value of 1.0 × 10^−5^. Nonpeptidase homologue proteins were excluded. Transmembrane transporters were predicted from nonsecreted proteins with TransporterTP server (http://bioinfo3.noble.org/transporter/) [51] using a cut-off e-value of 1.0 × 10^−3^. Genes associated with the synthesis of secondary metabolites were identified using Smurf (http://smurf.jcvi.org/index.php) [52,53] with default parameters.

The prediction of other protein sequences was performed using Blast2GO, which attributed Gene Ontology (GO) term, Interpro number and PFAM number to genes that showed conserved domains. Different families of transcription factors were identified based on conserved domains. In addition, Necrosis and Ethylene-inducing Proteins (NEPs) were predicted using conserved domains specific to NEP-like proteins (IPR008701/PF05630) [54]. Cytochrome P450 were identified via the family conserved domain (PF00067) [55]. Nudix proteins were identified by a family conserved domain (PF00293; IPR015797) [56]. The remainders were manually classified in putative functional groups according to Hmmscan, BLASTP and based on their predicted function. It is noteworthy that a pathogenicity-related class was created which included genes involved in stress responses and other pathogenicity factors such as genes involved in toxin synthesis. Nudix and NEP were further included in this class while cytochrome P450 genes were included in the oxidoreduction class. Proteins without predicted function, without predicted conserved domain, or with contradictory prediction were characterized as hypothetical proteins.

All predicted functions were further validated using Hmmscan program and Pfam database (version 33.0) with a cut-off e-value of 1.0 × 10^−3^, and a BLASTP searches to the GenBank nr database (release 233) using an e-valuethreshold of 1.0 × 10^−5^.

The agriGO v2.0 platform [57] was used to perform a customized Singular Enrichment Analysis (SEA) aiming to identify enriched GO terms in the up- and down- regulated DEGs, and in the detected proteins. Statistical analysis concerning RNAseq was performed using the predicted *C. lupini* proteome as reference on a query gene set which included genes that were up- or down-regulated in infected plants compared to the liquid culture condition. The percentage of DEGs in each GO category was calculated compared to the total of genes containing the same GO term in RB221 genome. Enriched GO terms were detected using the Fisher’s exact test (multi-test adjustment method: Hochberg, FDR = 0.05).

## 3. Results

### 3.1. Symptom Evaluation and Morphological Structures

**Figure 1:**
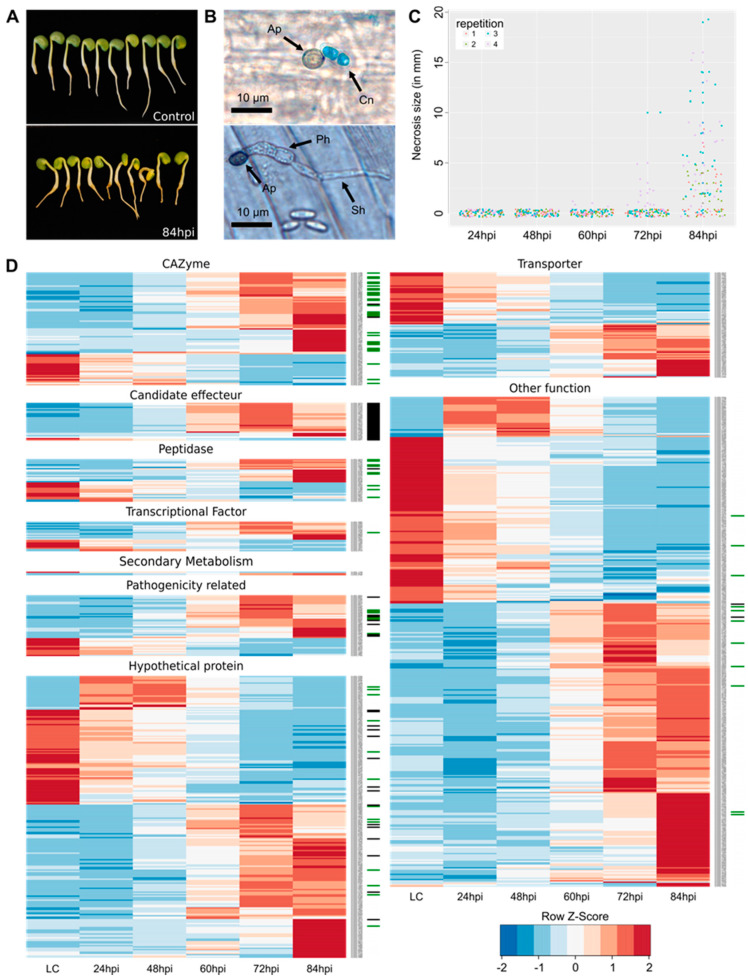
Overview of the phenotypic and molecular *C. lupini* infectious process. (**A**) Lupin inoculated with water (top) and with RB221 (bottom) at 84 hpi. (**B**) Germinated conidia (Cn) of the strain RB221 on lupin epidermis with appressorium (Ap) at 12 hpi (top) and formation of a primary hyphae (Ph) and a thinner secondary hyphae (Sh) at 48 hpi (bottom). Fungal structures were colored by lactophenol cotton blue and the length of the scale bar is 10 µm. (**C**) Evolution of the size of necrosis caused by *C. lupini* on lupin hypocotyl for 84 hpi. (**D**) Heatmap of 897 *C. lupini* DEGs compared to liquid culture (LC) (LFC < −1 and > 1, FDR < 0.05), grouped by putative functional class. Pathogenicity-related genes included stress response genes, Nudix, NEP and other functions described as associated with pathogenicity. Colored bars indicated gene expression by Z-score calculated from normalized reads of DEGs, and ranging from −2 (downregulated gene, blue) to 2 (upregulated gene, red). The green or black rectangles at the right of each gene represent genes respectively predicted as encoding secreted proteins or small secreted proteins.

The inoculation of lupin radicle with *C. lupini* first induced stem and petiole twisting as early as 60 hpi. Necrosis symptoms around the inoculation point began to appear at 60 and 72 hpi on a few plants, before being clearly visible at 84 hpi on 65% of plants (Figure 1A,C). Necrosis size measured at 84 hpi significantly varied according to biological repetition (*p* = 1.36 × 10^−5^). Appressoria were visible on epidermis of lupin hypocotyls as early as 12 hpi, and infection structures, like primary and secondary hyphae, were further observed at 48 hpi (Figure 1B).

### 3.2. Transcriptomic and Proteomic Data and Selection

Transcriptomic analysis was performed on RNA extracted from a liquid culture of *C. lupini* RB221, and from lupin hypocotyl inoculated with the same strain at 24, 48, 60, 72 and 84 hpi. For each sample, the total number of reads varied between 50.6 M and 85.5 M. Between 94.83% and 98.41% of total read pairs were kept after the cleaning step performed with Trimmomatic. Of these, 83.19% to 93.14% were paired and kept for further analysis (Table S1). The average mapping rate for the reads from RB221 liquid culture growth was 84.91%. The average mapping rate from infected plants ranged from 0.18% at 24 hpi to 3.54% at 84 hpi (Table S1).

With the aim of identifying *C. lupini* proteins produced during the onset of lupin infection, total proteins were extracted from lupin plants at 36, 60, 72, 84 and 96 hpi with RB221. Proteins were then identified and quantified by nLC Q exactive orbitrap MS-MS. Peptides quantified based on XICs led to the identification of 4377 putative proteins of both *C. lupini* and *L. albus*. Of these, 304 were identified and associated with *C. lupini* after filtering (below named QPs for quantified proteins) (Table S2). 

### 3.3. Overview of Gene Expression and Protein Synthesis

**Figure 2:**
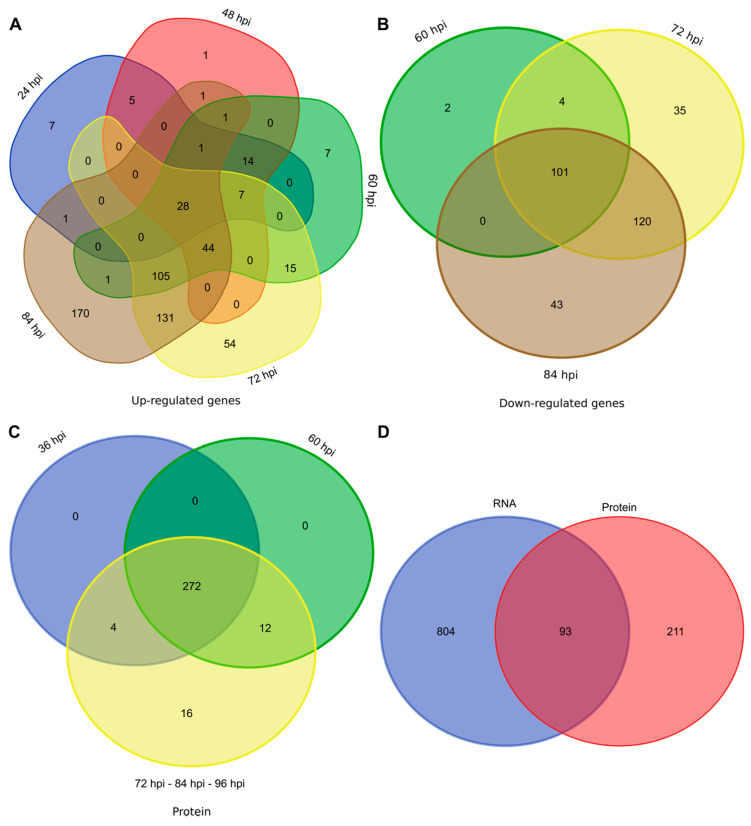
Venn diagram showing overlap of the 593 upregulated genes (**A**), 304 downregulated genes (**B**) and 304 QPs (**C**) across infection stages, together with the overlap of genes and proteins based on results from both transcriptomic and proteomic analysis during infectious process (**D**).

Compared to in vitro liquid cultures, 897 genes were differentially expressed by *C. lupini* RB221 in planta for at least one sampling time. Among these genes, 593 were upregulated (Figure 2A and Table S3) and 304 were downregulated (Figure 2B and Table S3) compared to the liquid culture condition. At 24, 48, 60, 72 and 84 hpi, 63, 103, 223, 384 and 483 genes were respectively upregulated. In addition, 7, 1, 7, 54, and 170 genes were uniquely upregulated at 24, 48, 60, 72 and 84 hpi, respectively (Figure 2A). In total, only 16 genes were uniquely expressed in planta (i.e., there were no counts in the in vitro condition, and more than one read for at least three out of the four repetitions in at least one sampling time), most of which encoding hypothetical proteins.

Upregulated genes compared to liquid cultures were detected from 24 to 84 hpi, while downregulated genes were detected later, from 60 to 84 hpi (Figure 2A,B).

For protein analysis, samples were collected 12 h after those for RNA analysis, to take into account the duration of translation steps (i.e., 36, 60, 72, 84 and 96 hpi). In total, 272 proteins from the 304 QPs were detected from 36 to 96 hpi. Among them, 16 were specific to the later stages, from 72 to 96 hpi (Figure 2C). For 99.3% of the QPs, the highest abundance level was reached at the last sampling time, i.e., 96 hpi.

Among the 304 identified proteins, only 93 corresponded to DEGs identified in the transcriptomic approach (Figure 2D). 

**Figure 3:**
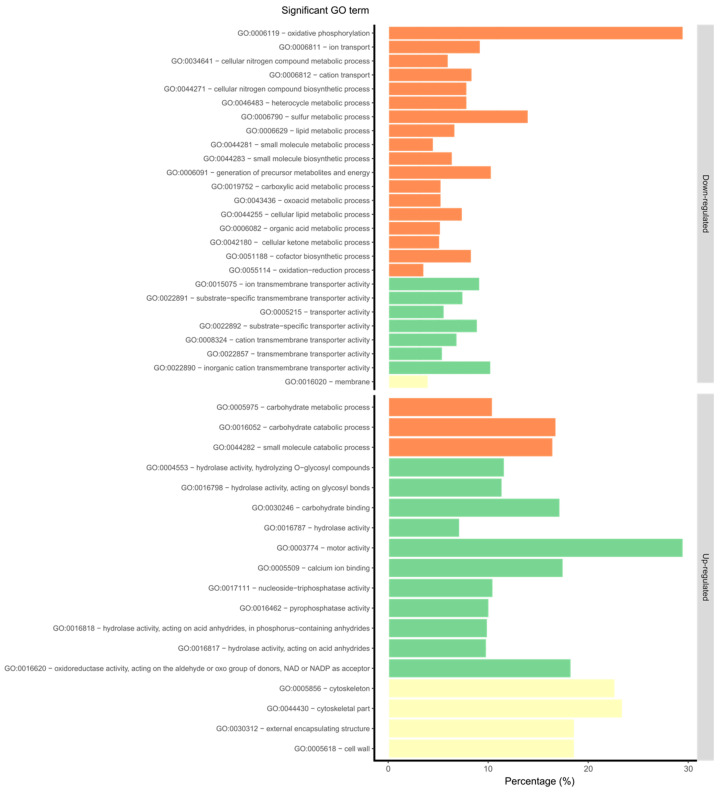
Significantly-enriched GO terms obtained from the GO enrichment analysis of all DEGs. The three main GO categories represent biological processes (orange), molecular functions (green), and cellular components (yellow). For each GO term, the percentage of up- and down-regulated genes compared to the number of genes in the genome associated with the same GO term is represented. Significant GO terms associated with DEGs were ordered by increasing FDR value.

By Blast2GO analysis, a total of 7132 genes with at least one GO term were found in the RB221 predicted proteome. Among the lists of DEGs and QPs, at least one GO term could be attributed to 331 upregulated genes, 163 downregulated genes and 245 proteins. Of these, respectively 18 and 26 GO terms were significantly enriched in the up- and down- regulated gene lists, as well as and 142 among QPs, respectively (Figure 3).

Among the upregulated genes, the most significant GO terms related to biological processes, molecular functions and cellular components were respectively associated with the carbohydrate catabolic process, motor activity and cytoskeleton modifications. Among downregulated genes, they were respectively associated with oxidative phosphorylation, transporter activities and membrane modifications.

### 3.4. Expression of the Predicted Functional Class of DEGs and QPs in Lupin Infection

#### 3.4.1. Secreted Proteins, Small-Secreted Peptides (SSP)

Secreted proteins constitute mobile molecules involved in pathogenicity which can directly interact with the host. The transcriptomic and proteomic analysis respectively showed that the secretion of 103 DEGs and 27 QPs was predicted. Twenty six out of these secreted proteins were identified by both analyses. They contained a signal peptide, were localized in the extracellular space and had neither a TMH domain nor a GPI anchor; these three criteria were used as a definition of secreted proteins by Zhang et al. (2018) [12] (Table 1). The number of secreted proteins from the DEG list was narrowed down to 46, which had less than 300 amino acid residues; of these, eight were also found in the QP list (Table 1).

#### 3.4.2. Candidate Effectors

**Figure 4:**
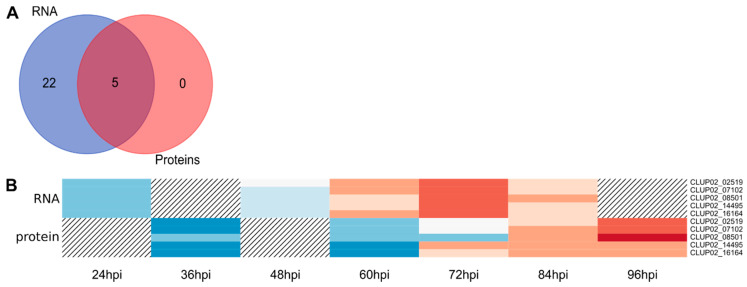
Overview of *C. lupini* expression and synthesis of candidate effectors. Venn diagram showing the overlap between candidate effector-encoding genes upregulated compared to liquid cultures in the RNAseq study and the candidate effector proteins revealed by this study (**A**). Heatmap of the five quantified proteins associated with candidate effectors function, regarding their abundance and the corresponding transcript expression profile. Hatching areas indicate that data are not available (**B**). Colored bars indicated gene expression by Z-score calculated from normalized reads, and ranging from −2 (downregulated gene, blue) to 2 (upregulated gene, red), or protein abundance by Z-score calculated from protein log10 abundance, and ranging from −2 (lowest abundance, blue) to 2 (highest abundance, red).

Effectors are SSP that enable the pathogen to manipulate host cell metabolism and overcome defense responses [58]. Among SSPs, 27 DEGs were predicted as effectors, among which, five were also found in the QP list (Figure 4A and Table 2). The analysis of *C. lupini* genome led to the identification of 973 genes encoding secreted proteins, among which 294 were identified as candidate effectors, indicating that 9.1% of total candidate genes encoding effectors were differentially expressed from control conditions and 1.7% were proteins identified during the first 84 hpi. Among the candidate effectors predicted from the DEG list, five contained a putative conserved hypothetical protein domain also detected by mass spectrometry (CLUP02_14495), a LysM domain (CLUP02_16647), a ToxB N-terminal domain (CLUP02_01667), a fungal hydrophobin domain (CLUP02_07198) or a meiotically upregulated gene family domain (CLUP02_12726). One candidate effector (CLUP02_16164) matched with a *Colletotrichum* spp. hypothetical protein (from 95.6% to 97.3% of identity, e-value = from 6.0 × 10^−60^ to 2.0 × 10^−75^) but also with a putative secreted-in-xylem 11 (SIX 11) effector of *Fusarium oxysporum* in the second best result (79.28% identity, e-value = 8.0 × 10^−60^) [59]. This candidate effector was also identified in the QP list. Most candidate effectors were upregulated compared to liquid cultures from 60 hpi to 84 hpi (Figure 1D) and the number of upregulated candidate effectors increase strongly between 24 hpi and 48 hpi (Table 2). Five candidate effectors were identified by mass spectrometry, of which three were detected as early as 36 hpi, and their abundance increased steadily to 96 hpi. CLUP02_16164 and CLUP02_14495 were detected only after 72 hpi (Figure 4B).

#### 3.4.3. Carbohydrate Active Enzymes (CAZymes)

**Figure 5:**
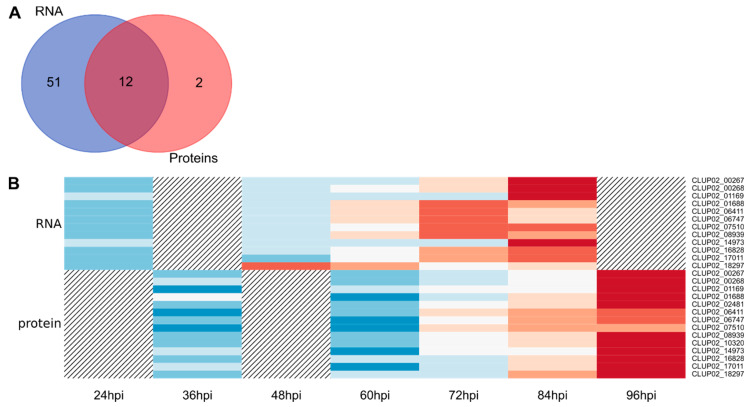
Overview of *C. lupini* expression and synthesis of candidate CAZymes. Venn diagram showing the overlap between CAZyme-encoding genes upregulated from RNAseq analysis and the candidate CAZymes proteins revealed by proteomic analysis (**A**). Heatmap of the 14 quantified proteins associated with CAZyme function, regarding their abundance and the corresponding transcript expression profile. Hatching areas indicate that data are not available (**B**). Colored bars indicated gene expression by Z-score calculated from normalized reads, and ranging from −2 (downregulated gene, blue) to 2 (upregulated gene, red), or protein abundance by Z-score calculated from protein log10 abundance, and ranging from −2 (lowest abundance, blue) to 2 (highest abundance, red).

CAZymes play an important role in many carbohydrate metabolism processes, some of which are related to host pathogenicity as degrading or binding enzymes to the host plant cell wall. A total of 63 candidate CAZymes were detected from the DEG list, 14 from the QP list and 12 were shared by both analyses (Figure 5A). These CAZymes belonged to six classes: 13 associated with auxiliary activities, 38 to glycosyl hydrolases including one associated with a starch binding domain (CBM20) and another associated with a polysaccharide deacetylase (CE4), seven to glycosyl transferases, one polysaccharide lyases, five to carbohydrate esterases and one to carbohydrate-binding modules (Table 2). The analysis of *C. lupini* genome led to the identification of 798 CAZyme-encoding genes, indicating that 7.9% of total CAZyme-encoding genes were differentially expressed compared to control conditions. Among the 65 predicted CAZymes, 28 were secreted proteins (including six from the QP list) and two were small-secreted proteins. The DEGs associated with CAZymes were mostly upregulated in the late stage of the infection, including a set of 23 genes specifically upregulated 84 hpi. Among significantly downregulated genes, downregulation was also more intense after 72 hpi (Figure 1D). Regarding CAZymes identified by mass spectrometry, all, except for CLUP02_07510 (which was detected later from 72 hpi to 96 hpi), were detected from 36 hpi to 96 hpi, and their abundance increased throughout the infection process (Figure 5B). Identified CAZymes were mainly involved in degradation of fungal or plant cell wall, glycosylation of proteins, energy, storage and recovery or synthesis of fungal cell wall (Table 3).

#### 3.4.4. Peptidases

**Figure 6:**
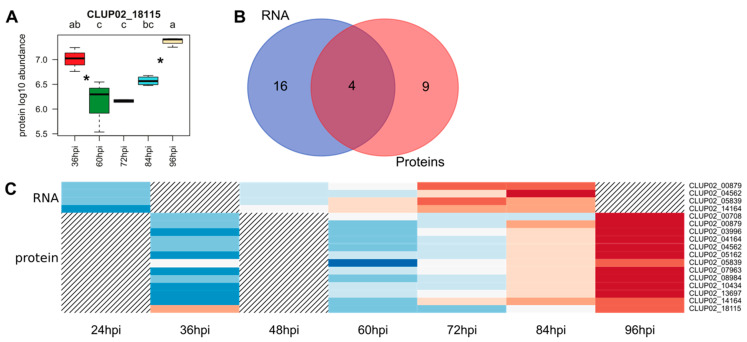
Overview of *C. lupini* expression and synthesis of candidate peptidases. Boxplot showing the mean abundance of the candidate peptidase from four biological repetitions and quantified by mass spectrometry (**A**). Venn diagram showing the overlap between peptidase-encoding genes upregulated in the RNAseq analysis and the peptidase proteins revealed by proteomic analysis (**B**). Heatmap of the 13 candidate peptidases regarding their abundance and their corresponding transcript expression profile. Hatching areas indicate that data are not available (**C**). Colored bars indicated gene expression by Z-score calculated from normalized reads, and ranging from −2 (downregulated gene, blue) to 2 (upregulated gene, red), or protein abundance by Z-score calculated from protein log10 abundance, and ranging from −2 (lowest abundance, blue) to 2 (highest abundance, red). Asterisks between histograms indicate significant changes over time.

Peptidases also contribute to the degradation of host proteins and are involved in pathogenicity. The analysis of *C. lupini* genome led to the identification of 504 peptidases and 25 peptidase inhibitor-encoding genes. During the interaction with lupin, 20 DEGs and 13 QPs, among which four were identified by both analyses, were predicted as peptidases (Figure 6B). The DEGs associated with peptidase activity included one peptidase inhibitor as well as 11 serine peptidases, three cysteine peptidases, 11 metallopeptidases, two threonine peptidases and one ubiquitin protease, among which seven were predicted as secreted (Table 2). The upregulated putative peptidases and the peptidase inhibitor were mostly upregulated after 72 hpi, except for CLUP02_12375, a putative cysteine peptidase mainly upregulated as early as 24 hpi compared to liquid cultures (Figure 1D), and for CLUP02_18115, a putative metallopeptidase quantified as early as 36 hpi (Figure 6C) (Table 2). All proteins were detected as early as 36 hpi and their abundance increased to 96 hpi except for CLUP02_18115, a putative metallopeptidase family M3. The abundance of the latter protein decreased significantly between 36 and 60 hpi before increasing again from 72 to 96 hpi (Figure 6A,C).

#### 3.4.5. Transmembrane Transporters

**Figure 7:**
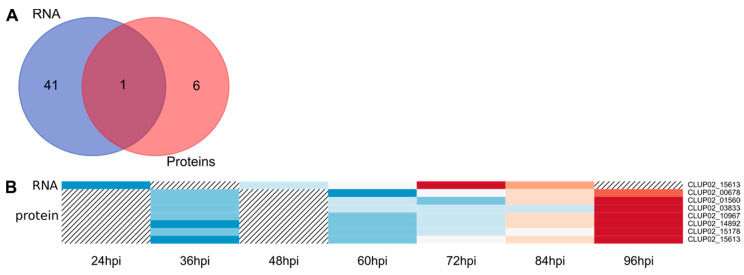
Overview of *C. lupini* expression and synthesis of transmembrane transporters. Venn diagram showing the overlap between DEGs and QPs associated with transmembrane transporter functions (**A**). Heatmap of the 7 candidate transmembrane transporters regarding their protein abundance and the corresponding transcript expression profile. Hatching areas indicate that data are not available (**B**). Colored bars indicated gene expression by Z-score calculated from normalized reads, and ranging from −2 (downregulated gene, blue) to 2 (upregulated gene, red), or protein abundance by Z-score calculated from protein log10 abundance, and ranging from −2 (lowest abundance, blue) to 2 (highest abundance, red).

During plant-pathogen interaction, transmembrane transporters are actively deployed for the uptake of carbohydrates, oligopeptides, ions and other molecules released by plant cell lysing. The analysis of *C. lupini* genome led to the identification of 884 transmembrane transporter-encoding genes. By transcriptomic and mass spectrometry analyses, respectively 41 (4.6% of total transmembrane transporters in the genome) and seven putative transmembrane transporters were identified, among which only one was detected by both analyses (Figure 7A). The transcripts belonged to 16 transporter families, including 14 belonging to the Major Facilitator superfamily (MFS), seven to the ATP-binding Cassette (ABC) superfamily and five to the P-type ATPase (P-ATPase) superfamily (Table 2). From 11 to 31 DEGs encoding transmembrane transporters were upregulated compared to liquid cultures from 60 hpi onwards while detected from 48 hpi. Downregulation of genes encoding transmembrane transporters was more intense in the late stages of the infection (Figure 1D). All seven proteins were detected as early as 36 hpi and their abundance increased until 96 hpi (Figure 7B).

#### 3.4.6. Genes Associated with Secondary Metabolism

**Figure 8:**
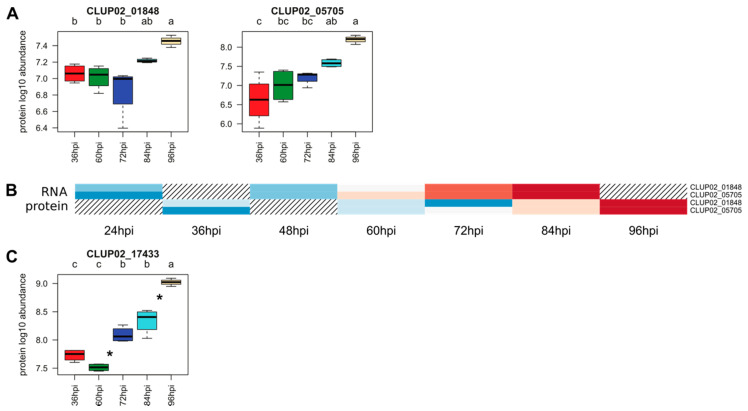
Overview of *C. lupini* expression and synthesis of genes and proteins associated with secondary metabolism and transcription factors. Boxplots showing the mean abundance of proteins associated with secondary metabolism from four biological repetitions and quantified by mass spectrometry. (**A**). Heatmap of the two proteins associated with secondary metabolism regarding their abundance and the corresponding transcript expression profile. Hatching areas indicate that data are not available (**B**). Boxplots showing the mean abundance of the transcription factor from four biological repetitions quantified by mass spectrometry. (**C**). Colored bars indicated gene expression by Z-score calculated from normalized reads, and ranging from −2 (downregulated gene, blue) to 2 (upregulated gene, red), or protein abundance by Z-score calculated from protein log10 abundance, and ranging from −2 (lowest abundance, blue) to 2 (highest abundance, red). Asterisks between histograms indicate significant changes over time.

The analysis of *C. lupini* genome led to the identification of 56 genes associated with secondary metabolism. Within the DEG and QP lists, only two secondary metabolism associated genes were predicted, being 3.6% of total genes associated with secondary metabolism in the genome (Figure 8B). CLUP02_01848 and CLUP02_05705 were characterized by multiple conserved domains such as polyketide synthase dehydratase, condensation domain, phosphopantetheine attachment site and ketoreductase domain (KR; Table 2) and were both identified as hybrid (NRPS/PKS1). CLUP02_05705 shared 44.3% identity with a *Beauveria bassiana* Tenellin synthetase (e-value = 0), a protein involved in the pathogenicity of this fungus against insects [67] and 37.7% identity with an *Aspergillus flavus* hybrid PKS-NRPS synthetase (e-value = 0) that mediates biosynthesis of leporins, a precursor of toxins such as aflatoxins [68]. Likewise, CLUP02_01848 shared 39.7% of identity with *Aspergillus clavatus* polyketide synthase-nonribosomal peptide synthetase (e-value = 0) involved in mycotoxin biosynthesis [69]. Neither of these two genes was specific to any infection stage, but the maximum level of expression was reached at the end of the kinetics (Table 2). The same proteins were detected from 36 to 96 hpi, but no significant increase was noticed over time (Figure 8A).

#### 3.4.7. Transcription Factors

Based on the analysis of conserved domains, 489 genes encoding transcription factors were predicted in the entire genome. A total of 16 DEGs (3.3% of total genes encoding transcription factors) and one QP were identified as transcription factors distributed in eight families (Table 2). Four putative transcription factors contained conserved domains including a fungal specific transcription factor with a Zinc finger C2H2 type or a fungal Zn(2)-Cys(6) binuclear cluster domain. One was predicted as secreted (CLUP02_11984). Most DEGs encoding candidate transcription factors were upregulated compared to liquid culture starting at 60 hpi, except two genes which were mainly over-expressed at 24 hpi followed by a significant decrease of expression between 60 and 72 hpi (CLUP02_01379 and CLUP02_02013; Figure 1D). The only protein identified as a transcription factor (CLUP02_17433) belonged to the TFIID complex and was detected from 36 to 96 hpi and its abundance increased after 60 hpi (Figure 8C).

#### 3.4.8. Cytochromes P450

Within *C. lupini* genome, 228 genes contained a cytochrome P450 conserved domain, and 11 were identified in the DEG list suggesting that 4.8% of the 228 genes were differentially expressed during the first 84 hpi in planta (Table 2). Putative functions were attributed to four of them, being L-ascorbate oxidase, ent-kaurene oxidase, benzoate 4-monooxygenase and N-alkane-inducible cytochrome P450. The 11 genes were mainly upregulated between 72 and 84 hpi, but one gene (CLUP02_02376) was significantly downregulated between 72 and 84 hpi. No cytochrome P450 was detected by mass spectrometry analysis.

#### 3.4.9. Factors Associated with Pathogenicity and Stress Response

**Figure 9:**
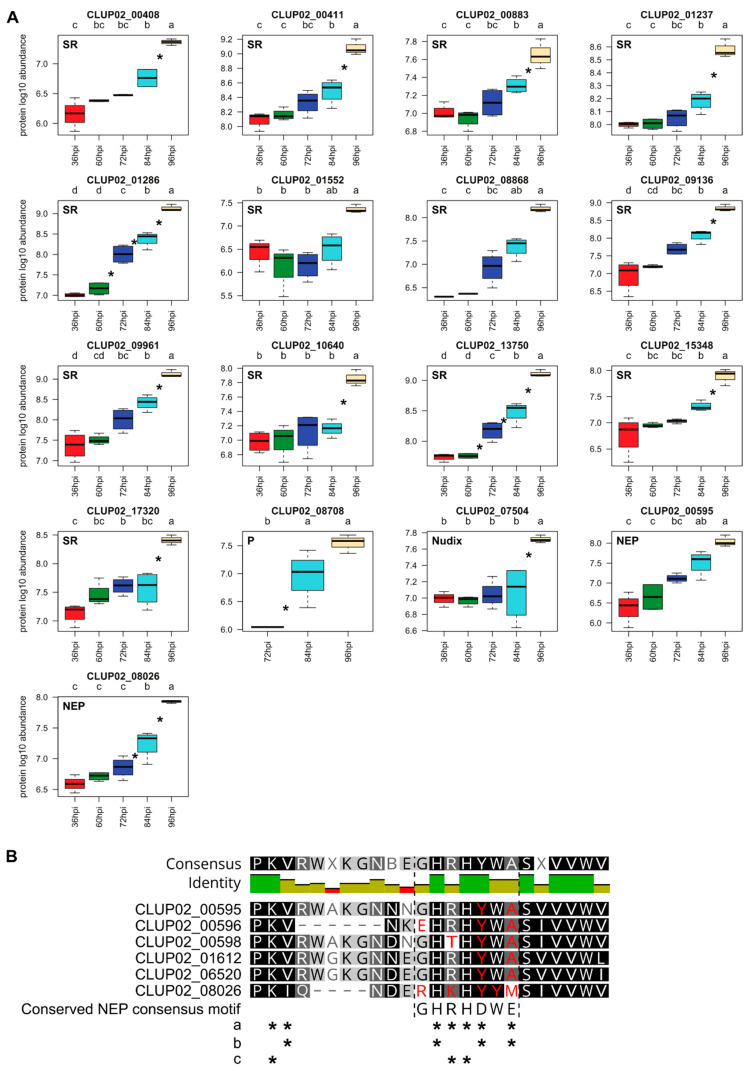
Abundance of representative pathogenesis-related proteins from *C. lupini* (Nudix, NEP, stress responses (SR) and other pathogenicity proteins (P)) across the infection kinetics. Boxplots show the mean abundance of pathogenesis-related proteins from four biological repetitions quantified by mass spectrometry. Asterisks between histograms indicate significant changes over time (**A**). Alignment of the protein sequences of the six NEPs differentially expressed in *C. lupini* during infection. The conserved amino acid residues were represented in green on the identity bar. Residues different from the conserved NEP pattern were identified in red. Asterisks indicate residues characterized as crucial for NEP activity (a), amino acid residues by alanine replacements resulting in abolished (b) or reduced (c) activity [14,73] (**B**).

Additional factors were detected by both RNA sequencing and mass spectrometry analyses and identified as involved in pathogenicity but did not belong to the previous functional class described earlier, including putative Necrosis and ethylene-inducing proteins (NEP), Nudix and stress responses proteins. NEPs, poorly studied in *Colletotrichum* spp., are known to elicit plant defenses, especially triggering hypersensitive response in dicots [70]. In *C. lupini* genome, 18 NEPs were identified of which six were upregulated during the infection and of these, two were detected by mass spectrometry (CLUP02_00595 and CLUP02_08026). They were all secreted and two of them (CLUP02_00596 and CLUP02_06520) were small-secreted peptides. The upregulation of NEPs was specific to medium infection stages since the maximum number of upregulated genes encoding NEPs was reached at 60 hpi (Table 2). Synthetized NEPs were detected as early as 36 hpi and their abundance increased until 96 hpi (Figure 9). The six NEPs we identified showed mutations in the pattern “GHRHDWE” especially “D” and “E” which are crucial for the NEP activity suggesting that the NEP activity of these proteins was abolished [14] (Figure 9B). CLUP02_13373 was identified as a putative intracellular hyphae protein. It contained a LysM domain probably involved in host interaction for establishment and maintenance of biotrophy, prevention of host recognition of the fungus and a barrier to host defense molecules [14,71,72].

Nudix were previously described as proteins belonging to effectors and potential markers of the end of the biotrophic phase and the start of the necrotrophic phase for hemibiotrophic pathogens [56]. In the entire genome of *C. lupini*, 20 genes were predicted as Nudix. Two genes encoding Nudix proteins were upregulated between 72 and 84 hpi (Table 2), among which one was also detected by mass spectrometry (CLUP02_07504) at 36 hpi and the highest amount of this protein was reached 96 hpi (Figure 9A). CLUP02_07504 was predicted as a secreted protein. Among fungal stress response proteins, 16 genes were detected in the DEG list and 13 stress response proteins in the QP list, four were detected during infection by both analyses, all predicted as heat shock protein. The abundance of most stress response QPs was significantly higher at the end of the kinetics (i.e., 11/13 with significantly higher amounts at 96 hpi; Figure 9A). Then, other factors were predicted with function associated with pathogenicity, like protein with CFEM domain, *Alternaria alternata* allergen, cerato platanin. Among them, nine genes encoding proteins related to pathogenicity were upregulated and whose one was detected by mass spectrometry (CLUP02_08708) (Figure 9A). Respectively, two, one and two genes were significantly upregulated between 24 and 48 hpi, between 48 and 60 hpi and between 72 and 84 hpi. The only protein detected was quantified from 72 to 96 hpi and its abundance significantly increased between 72 and 84 hpi (Figure 9A).

#### 3.4.10. Other Functions

Among the 593 upregulated DEGs, 213 were classified in the families described above, i.e., CAZymes, candidate effectors, transcription factors, secondary metabolism enzymes, transporters, peptidases, NEPs, Nudix, stress response, pathogenicity related and cytochrome P450. Most of the remainder genes (230) were upregulated between 60 and 84 hpi while a smaller part (40) was upregulated earlier between 24 and 48 hpi. These early-expressed genes were mainly involved in binding, cell structure, transcription, translation and oxidoreduction activities. Furthermore, 144 putative hypothetical proteins were identified, among which 17 were predicted as secreted proteins and seven were small-secreted proteins. Most hypothetical proteins were also upregulated between 60 and 84 hpi, except 26 which were mainly expressed between 24 and 48 hpi (Figure 1D and Table 2). In the QP list, 215 proteins were identified with other putative functions especially proteins related to oxidoreduction activities (42 proteins), translation (48 proteins) mainly represented by ribosomal proteins and binding (28 proteins). Thirteen hypothetical proteins were found, among which eight were assigned as hypothetical by blast and 22 resulted from contradictory or incompatible predictions of functions depending on protein function assignment tools.

## 4. Discussion

*C. lupini* is a major threat for lupin crops and control solutions need to be found in order to use this leguminous crop in rotations as a promising alternative for protein sources. A few studies have been performed to identify its taxonomy through phylogenetic analysis [74,75,76,77,78], describe its morphology [75,77,78] and symptoms in the field [4] or evaluate the effect of temperature on its in vitro and in planta growth [78,79,80]. In this study, Illumina RNA sequencing and nLC mass spectrometry were used to identify candidate genes and proteins associated with *C. lupini* pathogenicity over the course of infection in lupin plants. Analyses were performed on living lupins inoculated with the pathogen and associated with symptom evaluation. We chose to work on plants inoculated at a pre-seedling stage, and not on detached organs, since detachment may also represent stressful conditions for the plant, ultimately altering fungal responses during host colonization. In addition, the inoculation was performed on the radicle emerging from the seed in order to mimic the primary inoculum source in fields where infected seeds are planted. The deciphering of the first steps of the infection by other *Colletotrichum* species has already been performed, e.g., for *C. higginsianum* on *Arabidopsis* [11], *C. falcatum* on sugarcane [13], *C. fruticola* on strawberry [12] or *C. lentis* on lentil [56]. Among these, both shared and species-specific molecular patterns of infection were reported. In this study, 897 DEGs were found in planta at 24, 48, 60, 72 and 84 hpi compared to *C. lupini* liquid cultures (filter of LFC > 1 and LFC < −1) while 304 proteins could be quantified in planta at 36, 60, 72, 84 and 96 hpi. Candidate functions were associated with some of these genes such as CAZymes, transmembrane transporters, peptidases, candidate effectors, transcription factors, secondary metabolism and cytochrome P450 which have already been associated with pathogenicity of *Colletotrichum* spp. in past transcriptomic analyses. For the first time, Nudix and NEPs known as pathogenicity markers were also reported here as one of the molecular mechanisms deployed during the infection, by both transcriptomic and proteomic analyses. Taken together, both analyses evidenced that only 93 genes and their corresponding proteins were differentially expressed during lupin infection. This low number of genes could be explained by the difference in kinetics applied for these analyses, i.e., protein analysis was performed 12 h after RNAseq analysis, and another limit is that the sampling for protein quantification ended at 96 hpi, when their abundance was still increasing. Futhermore, for both approaches, the drastic selection applied on data, and the different pipelines used may partially explain the low number of shared candidates, indicating also the reliability of this list of genes that may be involved in the pathogenicity of *C. lupini*. Below are described the putative molecular mechanisms used by *C. lupini* during the different stages of lupin infection based on transcriptomic and proteomic analyses.

During the early stage of infection, *Colletotrichum* spp. are known to develop an appressorium which enables to penetrate host cuticle and epidermal cells [81], the formation of appressoria is induced by molecular markers. In *C. orbiculare*, a signal transduction pathway led by cAMP dependent protein kinases induced spore germination, appressorium development and infection hyphae formation [81]. Similarly, putative transcripts encoding cAMP-dependent kinases were also identified as candidates for activation of melanin biosynthesis in *C. gloeosporioides* [9]. In this study, after 24 hpi, appressoria and then primary hyphae of *C. lupini* were observed by light microscopy. In addition, several DEGs associated with appressorium development were significantly upregulated at the early stages or downregulated at the end of the infection. Within our DEG list, a gene encoding a putative cAMP dependent protein kinase was identified with 88.22% similarity to a cAMP dependant candidate gene of *C. higginsianum* involved in appressorium production (e-value = 0) [11,19]. This gene was upregulated from 24 hpi to 60 hpi. In addition, a putative CAP protein, which is known to be involved in appressorium development, was identified as perilipin homolog [82] and was significantly downregulated at 72 and 84 hpi [82]. In addition, CLUP02_15638 identified as a putative CAS1 appressorium-specific protein (81% similarity to *C. higginsianum*; e-value = 2.0 × 10^−126^ [83]) was also found to be downregulated from 60 hpi to 84 hpi. Early stages are also associated with the production of adhesion proteins and hydrophobins which are extracellular membrane proteins necessary for the interaction with the environment and as such, play an important role in pathogenicity. They are also shown to act as a developmental sensor of hydrophobic surface and to be involved in appressorium formation, attachment of hyphae to hydrophobic surfaces and to influence hyphal wall composition (reviewed in [84]). During the infection of lupin by *C. lupini*, a putative hydrophobin was significantly downregulated from 60 to 84 hpi. Likewise, CFEM-domain proteins are extracellular membrane proteins which were only described in fungi, with a higher occurrence in pathogenic fungi, and are suggested to play an important role in promoting pathogenicity although the exact function is still unknown [85]. For instance, CFEM-domain containing proteins from *Magnaporthe grisea*, namely adenylate cyclase-interacting protein ACI1 and PTH11 proteins, were shown to be involved in appressorium development [86,87]. In *C. lupini,* two genes encoding a protein carrying a CFEM domain were uniquely upregulated either at 60 hpi or 84 hpi, with the latter sharing 79% identity with a putative adhesin Mad1 of *Colletotrichum* spp. (CLUP02_08454; e-value = 0). Taken together, these results also suggest that appressorium formation may still be at stake later during infection, notably for spores which may germinate later. While transcriptomic analysis provided clear evidence that the genes involved in appressorium formation were upregulated early during infection onset, we did not detect any protein to support this observation. Interestingly, at 24 hpi, a set of 27 DEGs encoding hypothetical proteins was upregulated, evidencing the need to further explore the functions of these genes which are likely to be necessary for the onset of infection. Among them, we found a putative ribosomal protein SL 7 (CLUP02_04935) and a putative conserved hypothetical protein (PF11309, CLUP02_02353).

At 48 and 60 hpi, the observation of secondary hyphae and the upregulation of genes encoding CAZymes, effectors and pathogenicity-related proteins in *C. lupini* indicates the beginning of host penetration and the start of the setup of the necrotrophic phase. This resulted in a general increase of abundance of the corresponding proteins until 96 hpi, as observed by proteomic dynamics. At these stages, the fungus was supposedly penetrating host cells while almost no necrosis symptoms were visible. Penetration inside host cells is supported by the expression of CAZymes that degrade cell walls. The detection of the corresponding genes began at 48 hpi (9 genes) with mainly glycosyl hydrolase families GH1, GH18, GH28, and GH13 and carbohydrate esterases including pectinesterases while CAZymes were generally detected and quantified as early as 36 hpi. In *C. fructicola*, genes encoding CAZymes were upregulated as early as 24 hpi [12]. In *C. lupini,* the number of upregulated CAZymes continued to increase at 60 hpi with a total number of 23 genes out of the 63 identified in the DEG list. Most of them belonged to the families GH28, GH30, GH31, GH37 and GH131, but also AA9 (formerly GH61), CE like pectinesterases and pectin lyases. Except for the upregulated putative chitin synthase (CLUP02_17349), all other CAZymes were associated with plant cell wall degradation. One CAZyme was identified as a putative glucose-methanol-choline (GMC) oxidoreductase, described previously in *Glomerella cingulata, C. higginsianum* and *C. graminicola* and other phytopathogenic ascomycetes as reductases of plant-produced anti-fungal quinones and phenoxy-radicals [88,89]. Early stages of plant penetration are also characterized by the induction of genes encoding effectors. For instance, in *C. higginsianum,* candidate effectors were mostly upregulated during the biotrophic phase at 40 hpi [11]. In *C. lupini*, we found only one DEG encoding a candidate effector (CLUP02_08501) upregulated at 24 hpi versus 11 at 48 hpi and 19 at 60 hpi. Among the genes encoding candidate effectors, some contained conserved domains associated with pathogenicity functions such as a lysin-motif domain (IPR018392/PF01476; CLUP02_16647) described as involved in the sequestration of chitin oligosaccharides to avoid recognition by the host and plant defense induction [90], or a fungal hydrophobin domain (PF06766) generally found on the outer surface of conidia and hyphal wall, and involved in mediating contact and communication between the fungus and its environment [91]. Among pathogenicity-related genes, 3 DEGs encoding NEPs were detected here and were expressed as early as 48 hpi and 6 were upregulated from 60 hpi onwards. This result is further supported by the presence of NEP proteins that were found to be increasingly synthesized from 36 hpi onwards. The switch between the biotrophic and necrotrophic phase of *C. higginsianum* in *A. thaliana* appeared between 40 hpi and 60 hpi [11]. Likewise, *C. fructicola* developed necrotrophic secondary hyphae on strawberry before 72 hpi [12]. Like NEPs, Nudix are considered as other putative markers of the biotrophy-necrotrophy switch and may induce hypersensitive responses of the host plant. This protein, notably described in *C. lentis* (previously identified as *C. truncatum*), contains a signal peptide and a Nudix hydrolase domain that may be unique to hemibiotrophic fungal plant pathogens [56]. Of the two Nudix-encoding genes reported in this study (CLUP02_07504), one was found to be upregulated at 60 hpi while the other was upregulated at 84 hpi, surprisingly the candidate Nudix protein encoded by CLUP02_07504 was detected as early as 36 hpi but was significantly produced in higher amounts 96 hpi. Taken together, these results may indicate a transition from biotrophy to necrotrophy around 48 hpi and are consistent with the observation of primary hyphae at that time.

Transmembrane transporters were also highly expressed at 60 hpi among which MFS, APC and ABC transporters were mainly represented while these proteins were detected between 72 and 96 hpi based on proteomic analysis. 

Overall, the setup of the necrotrophic phase seemed to occur around 60 hpi, based on our gene expression analysis. This was further corroborated by proteomic analysis, and also supported by observations on infected plants where necrotic lesions were almost absent at that time (only three hypocotyls out of 120 were found to display a 1 mm necrotic lesion) before clearly starting to appear at 72 hpi and keeping increasing at 84 hpi. Based on symptom observations, the necrotrophic phase was in progress between 72 hpi and 84 hpi. Among the DEGs, 54 genes were uniquely upregulated at 72 hpi and 168 at 84 hpi. These stages of infection, and even more at 84 hpi, were characterized by a general increase of the expression of genes encoding almost all families associated with the infectious process such as CAZymes, especially glycosyl hydrolases, peptidases, and transmembrane transporters, including sugar transporters (MFS), together with the general increase of the amount of corresponding QPs reaching a maximum 96 hpi. In contrast, the expression of most of the DEGs encoding candidate effectors remained stable or decreased significantly from 48 hpi onwards, except for 2 genes which expression was significantly increased from 72 and 84 hpi (CLUP02_01667 and CLUP02_12726). The former was identified as a putative ToxB-encoding gene, a toxin also produced by the rice blast fungus *Pyrenophora tritici-repentis* [92]. The latter gene contained two previously-described domains, the IPR015131/PF09044 domain identified as a toxin killer and the IPR029167/PF15474 involved in meiosis mechanism. These domains were already described among the predicted effectors of *Fusarium graminearum* [93,94]. Concerning CAZymes, 38 and 59 genes out of 63 were respectively upregulated at 72 and 84 hpi, mostly represented by glycosyl hydrolases and cellobiose dehydrogenases involved in plant cell degradation. Peptidases were also mainly expressed during the necrotrophic phase although detected in the transcriptomic analysis as early as 24 hpi. Likewise, proteomic analysis revealed that the highest levels of detected candidate CAZymes and proteases were reached 96 hpi. At the same time, genes encoding transmembrane transporters can be upregulated as they are required for assimilating the products of the degradative activity, as observed in *C. higginsianum* [11,12]. In particular, ABC and MFS transporters are known in other species to detoxify and eliminate antifungal secondary metabolites and various toxins produced by plants [95,96]. This function, which was characterized in *C. acutatum* CaABC1 transporter, may contribute to resistance against some fungicides, and was also necessary for conidiation and abiotic stress resistance [23], whereas some MFS transporters like Mfs1 in *C. lindemuthianum* are induced during the necrotrophic phase for mediating hexose uptake [97]. At this stage, genes encoding candidate enzymes involved in the response to oxidative stress were also upregulated, such as one catalase (CLUP02_03311) and two peroxidases (CLUP02_09271, CLUP02_09357). The expression of these genes in the late stages of the infection could indicate that *C. lupini* set up an alleviation of the oxidative stress, further contributing to detoxify the cell in response to the production of reactive oxygen species (ROS) such as hydrogen peroxide (H_2_O_2_) by the host [98,99]. Likewise, in *C. gloeosporioides* f. sp. *malvae*, a catalase-encoding gene was highly expressed in the necrotrophic phase of an infection of round-leaved mallow, *Malva pusilla* [100]. Last, another gene encoding a cerato-platanin protein putatively involved in pathogenicity was uniquely upregulated at 84 hpi, previously described as both an effector and an elicitor protein abundant in filamentous fungi. This dual role was evidenced in *C. truncatum* infecting many leguminous species, where the cerato-platanin protein was detected in the biotrophic phase as an effector suppressing plant defense responses, and may also be involved as an elicitor of plant defense responses in the necrotrophic phase [101,102]. In *C. falcatum*, one cerato platanin EPL1 was also found as a secreted protein eliciting systemic resistance in sugarcane and HR response in tobacco [103]. Likewise, in our study, the cerato-platanin-encoding gene (CLUP02_11344) seemed to be associated with the necrotrophic stage only.

This study proposes that the evaluation of the pathogenicity associated with a transcript and the proteome analysis approach can provide a better understanding of the infectious process of *C. lupini*. Taken together, these results suggest that *C. lupini* may be a hemibiotrophic fungal pathogen with a switch between the biotrophic and necrotophic phase probably occurring around 48 hpi under our experimental conditions. The first stages of the infection began with an appressorium development during the first 24 h post-inoculation as well as an increase of expression of genes involved in cellular processes. Some genes encoding hypothetical proteins were uniquely upregulated at the early stage of infection and their future identification will probably contribute to better understand the infectious process of the biotrophic phase. During the biotrophic-necrotrophic transition, expression of genes encoding proteins like NEP, Nudix, CAZymes, peptidases, transmembrane transporters and candidate effectors increased suddenly, this increase was consistent with the quantification of proteins from the same family and was associated with the apparition of necrotic lesions on infected plants. A few toxin-encoding genes were also upregulated in the necrotrophic phase. Overall, transcriptomic and proteomic analyses of *C. lupini* infecting lupins shed a new light on the putative mechanisms involved in the pathogenicity of *Colletotrichum* spp. over the course of the infection. However, the findings presented in this study need to be confirmed by functional experiments to validate the roles played in the infectious process.

## Figures and Tables

**Table 1:** Distribution of secreted proteins, small-secreted proteins and putative transmembrane proteins across infection stages for both transcriptomic and proteomic analysis.

	Transcriptomic Analysis						Proteomic Analysis				Shared between Methods
	24 hpi	48 hpi	60 hpi	72 hpi	84 hpi	Total Number of Transcripts	36 hpi	60 hpi	72/84/96 hpi	Total Number of Proteins	
Protein predicted with signal peptide—SignalP 5.0	9	37	72	110	119	135	37	37	40	40	31
Extracellular protein—WoLF PSORT	10	37	79	115	130	144	35	35	38	38	31
Transmembrane protein—TMHMM	14	15	40	77	86	118	21	21	21	21	6
Protein containing GPI anchor—PredGPI and GPI-som	3	3	9	13	18	21	8	8	9	9	3
Secreted protein (Extr, Signal P, no TMH, no GPI)	5	32	60	84	96	103	25	25	27	27	26
Small-secreted protein (secreted protein with size < 300 Amino Acid residues)	2	17	30	41	43	46	6	6	8	8	8
**Total of DEG or QP at each time point**	63	102	223	384	483	593	276	284	304	304	93

**Table 2:** Overview of the distribution of upregulated genes compared to liquid cultures across infection stages, according to the identification of conserved domains. *: those with one candidate effector.

Functional Class	Family	24 hpi	48 hpi	60 hpi	72 hpi	84 hpi	Total
Carbohydrate active enzyme	Auxiliary Activities *	0	1	5	7	13	13
	Glycoside hydrolases	0	6	11	22	33	37
	Carbohydrate binding modules	0	0	0	0	2	2
	Glycosyl Transferases	0	0	1	3	6	6
	Carbohydrate Esterases	0	3	6	6	6	6
	Polysaccharide Lyases	0	0	1	1	1	1
	Total of CAZyme domains	0	10	24	39	61	65
	Total of genes encoding CAZymes	0	9	23	38	59	63
Transcription factor	Fungal Zn(2)-Cys(6) binuclear cluster domain	0	0	2	2	4	4
	Fungal specific transcription factor	0	0	2	4	6	6
	Helix-loop-helix DNA-binding domain	0	0	0	0	1	1
	Zinc finger, C2H2 type	1	1	2	3	2	4
	bZIP transcription factor	0	0	0	1	2	2
	Spt20 family	1	0	0	0	0	1
	CP2 transcription factor	0	0	0	1	0	1
	Total of transcription factor domain	2	1	6	11	15	19
	Total of genes encoding transcription factors	2	1	4	9	12	16
Secondary metabolism	Polyketide synthase dehydratase	0	0	1	2	2	2
	Condensation domain	0	0	1	2	2	2
	Phosphopantetheine attachment site	0	0	1	2	2	2
	Ketoreductase domain (KR)	0	0	1	2	2	2
	Total of secondary metabolism domains	0	0	4	8	8	8
	Total of secondary metabolism associated genes	0	0	2	2	2	2
Candidate effector		1	11	19	26	24	27
Peptidase	Serine peptidase	1	2	3	6	9	10
	Metallopeptidase	0	1	2	3	6	6
	Cystein peptidase	2	1	1	0	3	3
	Peptidase inhibitor	0	0	0	0	1	1
	Total of genes encoding peptidases	3	4	6	9	19	20
Transmembrane transporter	The Ca^2+^:Cation Antiporter (CaCA) Family	0	0	1	4	0	4
	The Amino Acid/Auxin Permease (AAAP) Family	0	0	0	3	2	3
	The ATP-binding Cassette (ABC) Superfamily	0	1	2	4	4	5
	The Major Facilitator Superfamily (MFS)	0	0	3	7	13	14
	The Amino Acid-Polyamine-Organocation (APC) Family	0	0	3	2	3	4
	The Proton-dependent Oligopeptide Transporter (POT) Family	0	0	0	0	1	1
	The Small Conductance Mechanosensitive Ion Channel (MscS) Family	0	0	0	1	1	1
	The P-type ATPase (P-ATPase) Superfamily	0	0	1	3	2	4
	The Mitochondrial Carrier (MC) Family	0	0	1	1	1	1
	The Oligopeptide Transporter (OPT) Family	0	0	0	0	1	1
	The Transient Receptor Potential Ca^2+^ Channel (TRP-CC) Family	0	0	0	1	1	1
	Threonine/serine exporter	0	0	0	1	1	1
	Mitochondrial calcium uniporter	0	0	0	0	1	1
	Total of transmembrane transporter genes	0	1	11	27	31	41
Other pathogenicity related-factors	Nudix	0	0	1	1	2	2
	Necrosis and ethylene-inducing peptide *	0	3	6	6	6	6
	Stress response	0	0	3	12	13	16
	Pathogenicity	0	2	3	4	8	9
Oxydoreduction	Cytochrome P450	0	0	2	7	10	11
	NAD/FAD binding and other oxydo-reductase	3	8	17	37	53	58
Cellular process	AAA family	0	0	0	0	2	2
	Binding	6	7	14	15	10	24
	Cell differentiation	1	1	1	1	0	1
	DNA repair	1	1	1	1	1	1
	Intracellular transport	1	1	3	10	8	13
	Methyltransferase	1	1	3	6	10	13
	Mitochondria	1	1	2	2	1	2
	Protein kinase	1	1	2	3	6	7
	Transcription	4	3	3	6	4	8
	Translation	3	5	9	10	5	12
	Ubiquitinilation	1	1	1	2	4	4
Metabolic process	Alpha/beta hydrolase	0	0	1	1	2	2
	AMP-binding enzyme	0	0	1	1	2	2
	Carbohydrate metabolism	1	1	2	2	6	6
	Coenzyme A metabolism	0	0	0	0	1	1
	Lipid metabolism	0	0	0	4	5	6
Cell structure		4	4	6	9	6	11
Hypothetical protein		27	33	60	93	120	144
Others		2	3	18	40	51	63

**Table 3:** Overview of the specific functions of CAZymes encoded by DEGs or identified in *C. lupini* during interaction with white lupin.

Substrate	Number of Enzyme	CAZymes	Target					References
			Plant Cell Wall Degrading Enzyme	Fungal Cell Wall Degrading Enzyme	Energy Storage and Recovery	Protein Glycosylation	Fungal Cell Wall Synthesizing Enzyme	
Cellulose	1	AA3_1	X					[60,61,62]
	8	AA9	X					[60,61,62,63]
	1	GH7	X					[11,60,62,63,64,65,66]
Hemicellulose (Xyloglucan or Mannogalactan)	4	GH31	X		X	X		[11,60,61,62,63,64,65,66]
	2	GH35	X					[11,60,61,62,63,64,65,66]
Pectin	3	GH28	X					[11,60,61,62,63,64,65,66]
	1	GH88/GH105	X					[11,61,62,63,64,65]
	2	CE8	X					[11,61,62,63,65,66]
	1	PL1_4	X					[11,61,62,63,64,65,66]
Lignin	1	AA1	X					[60]
	1	AA3	X					[60]
	1	AA3_2	X					[60]
	1	AA3_3	X					[60]
Starch	1	CBM20						[63]
	1	GH15			X			[61,63,64,65]
Hemicellulose or Pectin	1	CE12	X					[11,60,61,62,63,65,66]
Hemicellulose (Xylan) or Chitin	1	CE4		X				[61,63,65]
	2	GH2	X	X				[60,61,62,63,64,65,66]
	4	GH3	X	X				[60,61,62,63,64,65,66]
Chitin or Cellulose	2	GH1	X	X				[60,61,62,63,64,65,66]
	1	GH5	X	X				[60,61,62,63,64,65,66]
	1	CBM33/AA10	X					[60,66]
Chitin	4	GH18		X				[61,63,64,65]
	1	GH76		X				[61,64,65]
	1	GT2					X	[61,63]
	2	GT2_Chitin_synth_2					X	[61,63]
Glucan (β-1,3-glucan)	1	GH131		X				[61,63]
	1	GH17		X				[61,63,65]
Glycoproteins (N-/O-glucans)	1	GH63				X		[61,64]
	1	GH47				X		[61,64]
Trehalose	1	GH37			X			[61,63,64]
Glycogen or Starch	1	GT35				X		[63]
-	1	GH92		X				[65]
	1	GT1						-
	2	GT2_Glyco_tranf_2_3						-
	1	GH30		X				[61,64]
	2	CE10						-

## Supplementary Materials

The following are available online at https://www.mdpi.com/2076-2607/8/10/1621/s1, Table S1: RNAseq read counts and percentage mapping statistics to *C. lupini* (RB221 strain) genome. Table S2: Log10 abundance of the 368 proteins. Abundance of proteins written in red have a *p*-value > 0.01 (64 proteins) and those written in green have a *p*-value < 0.01 (304 proteins). R1-4 corresponds to the number of the replicate. Table S3: Expression analysis for all *C. lupini* DEGs in comparison with liquid culture and measured during the infectious process using RNA-Seq. LC: Liquid Culture; IP: In planta; the number after LC or IP corresponds to time post-inoculation in hour.
