# Supplementary material for: Deciphering the Infectious Process of Colletotrichum lupini in Lupin through Transcriptomic and Proteomic Analysis
Source: Microorganisms. 2020 Oct 21;8(10):1621. doi: 10.3390/microorganisms8101621 (PMC7589765; doi:10.3390/microorganisms8101621)
Supplement: Supplementary file 1 [file microorganisms-08-01621-s001.zip › Table S1.docx]

Tab. S1: RNAseq read counts and percentage mapping statistics to *C. lupini* (RB221 strain) genome.

| Conditions | Hours post inoculation | Total reads | Clean reads | | Paired read | | Mapping rate of paired reads (%) |
| --- | --- | --- | --- | --- | --- | --- | --- |
|  |  |  | Number | % | Number | % |  |
| Liquid Culture | 24hpi | 75,369,910 | 73,081,970 | 100% | 63,814,239 | 87,32% | 84,91% |
| *In planta* | 24hpi | 61,619,009 | 60,468,982 | 100% | 55,520,419 | 91,82% | 0,18% |
|  | 48hpi | 64,075,213 | 62,854,616 | 100% | 57,850,622 | 92,04% | 0,19% |
|  | 60hpi | 54,031,295 | 53,030,718 | 100% | 48,765,725 | 91,96% | 0,30% |
|  | 72hpi | 64,460,832 | 63,274,378 | 100% | 58,284,321 | 92,11% | 1,02% |
|  | 84hpi | 63,280,187 | 61,863,914 | 100% | 56,303,120 | 91,01% | 3,54% |
